# Prevalence and incidence of anal high‐grade squamous intraepithelial lesions in a cohort of cisgender men and transgender women who have sex with men diagnosed and treated during acute HIV acquisition in Bangkok, Thailand

**DOI:** 10.1002/jia2.26242

**Published:** 2024-05-02

**Authors:** Supanat Thitipatarakorn, Nipat Teeratakulpisarn, Siriporn Nonenoy, Aphakan Klinsukontakul, Sujittra Suriwong, Jirat Makphol, Piranun Hongchookiat, Thanyapat Chaya‐ananchot, Napasawan Chinlaertworasiri, Pravit Mingkwanrungruang, Carlo Sacdalan, Kultida Poltavee, Tippawan Pankam, Stephen J. Kerr, Reshmie Ramautarsing, Donn Colby, Nittaya Phanuphak

**Affiliations:** ^1^ Institute of HIV Research and Innovation Bangkok Thailand; ^2^ SEARCH Research Foundation Bangkok Thailand; ^3^ Research Affairs Faculty of Medicine Chulalongkorn University Bangkok Thailand; ^4^ Thai Red Cross AIDS Research Centre Bangkok Thailand; ^5^ HIV‐NAT Thai Red Cross AIDS Research Center Bangkok Thailand; ^6^ Biostatistics Excellence Center Faculty of Medicine Chulalongkorn University Bangkok Thailand; ^7^ The Kirby Institute University of New South Wales Sydney New South Wales Australia; ^8^ Center of Excellence in Transgender Health Chulalongkorn University Bangkok Thailand

**Keywords:** HIV, precancerous conditions, human papillomavirus viruses, highly active antiretroviral therapy, sexual and gender minorities, early detection of cancer

## Abstract

**Introduction:**

Men who have sex with men (MSM), especially those living with HIV, are at an increased risk of anal cancer. The prevalence and incidence of its precursor, anal high‐grade squamous intraepithelial lesions (HSILs), among MSM who started antiretroviral therapy during acute HIV acquisition are yet to be explored.

**Methods:**

Participants in an acute HIV acquisition cohort in Bangkok, Thailand, who agreed to take part in this study, were enrolled. All participants were diagnosed and started antiretroviral therapy during acute HIV acquisition. Human papillomavirus (HPV) genotyping and high‐resolution anoscopy, followed by anal biopsy as indicated, were done at baseline and 6‐monthly visits.

**Results:**

A total of 89 MSM and four transgender women were included in the analyses. Median age at enrolment was 26 years. Baseline prevalence of histologic anal HSIL was 11.8%. With a total of 147.0 person‐years of follow‐up, the incidence of initial histologic anal HSIL was 19.7 per 100 person‐years. Factors associated with incident anal HSIL were anal HPV 16 (adjusted hazards ratio [aHR] 4.33, 95% CI 1.03–18.18), anal HPV 18/45 (aHR 6.82, 95% CI 1.57–29.51), other anal high‐risk HPV (aHR 4.23, 95% CI 1.27–14.14), syphilis infection (aHR 4.67, 95% CI 1.10–19.90) and CD4 count <350 cells/mm^3^ (aHR 3.09, 95% CI 1.28–7.48).

**Conclusions:**

With antiretroviral therapy initiation during acute HIV acquisition, we found the prevalence of anal HSIL among cisgender men and transgender women who have sex with men to be similar to those without HIV. Subsequent anal HSIL incidence, although lower than that of those with chronic HIV acquisition, was still higher than that of those without HIV. Screening for and management of anal HSIL should be a crucial part of long‐term HIV care for all MSM.

## 1 INTRODUCTION

Anal cancer is a rising concern for people living with HIV. The incidence of anal cancer among men who have sex with men (MSM) with HIV was 85 per 100,000 person‐years, a rate five times higher than MSM without HIV [1]. It is not mitigated by the use of antiretroviral therapy (ART) [2, 3]. Human papillomavirus (HPV) infection is the major cause of anal cancer [4, 5], with HPV 16 responsible for 70% of cases in MSM with HIV [4].

Anal high‐grade squamous intraepithelial lesions (HSILs) are the known precursors of anal cancer [6, 7]. Persistent HPV 16 is associated with persistent anal HSIL [8] and the greatest risk of anal cancer [8, 9]. Previous studies have shown differences in the prevalence and incidence of anal HSIL between people living with HIV compared to those without HIV [8, 9, 10, 11, 12]. The pooled prevalence of anal HSIL among MSM with HIV from a recent systematic review was 22.4%, twice as high as among MSM without HIV [12]. The incidence of anal HSIL among people living with HIV is also generally higher than that of people without HIV (10.5–31.3 vs. 4.9–10.1 per 100 person‐years) [8, 10, 11, 13, 14]. A recent study demonstrates that screening for and treating anal HSIL among MSM with HIV effectively prevents progression to anal cancer in almost 60% of cases [15].

Early ART initiation has been shown to preserve the immune system [16, 17, 18], but the prevalence and incidence of anal HSIL among those who initiate ART during acute HIV acquisition, before permanent immunologic damage, remains unknown. Therefore, the aim of this study is to describe the prevalence, incidence and associated factors related to anal HSIL progression among cisgender men and transgender women (TGW) living with HIV who began ART during acute HIV acquisition.

## 2 METHODS

### 2.1 Study design and population

This is a prospective observational cohort of cisgender men and TGW who have sex with men who initiated ART at the time of acute HIV acquisition diagnosis in the SEARCH010/RV254 cohort (ClinicalTrials.gov Identifier NCT00796146) in Bangkok, Thailand. Acute HIV acquisition was defined as Fiebig stages 1–5 [19] HIV acquisition. All participants were offered immediate ART. They were examined at baseline to determine the prevalence of anal HSIL and HPV infection within 1 week after ART initiation. They were then followed up every 6 months for the occurrence of anal HSIL over time. This study analysed data collected from participants enrolled from May 2017 to June 2020. Data from follow‐up visits until June 2021 were included in the analysis.

Data obtained at the baseline visit included demographic data, circumcision history, age of sexual debut, sexual behaviour, history of condom use, sexually transmitted infections, smoking, alcohol drinking and amphetamine‐type stimulant use. Laboratory tests, including sexually transmitted infection testing, CD4 count and plasma HIV‐1 RNA, were obtained from the SEARCH010/RV254 study database. Digital anorectal examination, anal cytology, HPV genotyping and high‐resolution anoscopy (HRA), followed by anal biopsy as indicated, were performed at the baseline and subsequent visits. This study analysed data collected from participants enrolled between May 2017 and June 2020. Data from follow‐up visits until June 2021 were included in the analysis.

All participants provided informed consent. The study was approved by the institutional review board of Chulalongkorn University in Bangkok, Thailand (IRB No. 706/59) and registered at ClinicalTrials.gov (Identifier NCT03032575).

### 2.2 Anal cytology, HRA and biopsy

Anal specimens were collected prior to HRA at each visit. A moistened, non‐lubricated polyester‐tipped swab was inserted 5–7 centimetres into the anal canal and slowly removed with a twirling motion and gentle pressure applied to the walls of the anal canal to maximize cellular yield. The swab was placed in a liquid‐based cytology fluid (ThinPrep®, Cytyc Corporation, Boxborough, MA, USA) for transport and storage until processing. HRA was performed at baseline and every 6 months by the same study physician (NT). Acetic acid solution and Lugol's solution were used to aid in the visualization of abnormal anal tissue. Anal biopsy was performed on suspicious lesions and processed and assessed by the pathology department of King Chulalongkorn Memorial Hospital. Cytology and histology results were reported in accordance with the terminology of the Lower Anogenital Squamous Terminology (LAST) Project [20]. Discrepancies were resolved by re‐evaluation, discussion and concurrence by at least two2 pathologists. Participants who had anal HSIL at their last study visits were offered treatment with infrared coagulation free of charge following the local standard of care.

### 2.3 HPV genotyping

HPV genotyping was performed on liquid‐based anal cytology fluid collected at baseline and every 6 months and stored at −80°C. HPV typing was done using the LINEAR ARRAY® HPV Genotyping Test (Roche Molecular Systems, Inc., New Jersey, USA). The tests amplified target DNA within the polymorphic L1 region of the HPV genome that is approximately 450 base pairs long by polymerase chain reaction. It then utilizes nucleic acid hybridization to independently identify 37 anogenital HPV DNA genotypes (6, 11, 16, 18, 26, 31, 33, 35, 39, 40, 42, 45, 51, 52, 53, 54, 55, 56, 58, 59, 61, 62, 64, 66, 67, 68, 69, 70, 71, 72, 73 [MM9], 81, 82 [MM4], 83 [MM7], 84 [MM8], IS39 and CP6108) in cells. These genotypes include the 14 high‐risk genotypes (16, 18, 31, 33, 35, 39, 45, 51, 52, 56, 58, 59, 66 and 68). To ensure proper extraction, amplification and cell adequacy, human β‐globin gene primers were used. Specimens negative for β‐globin amplification were excluded from analysis.

### 2.4 Laboratory testing

Testing for sexually transmitted infections, CD4 count and plasma HIV‐1 RNA was done as part of the SEARCH010/RV254 study. Syphilis testing was done with both treponemal (syphilis chemiluminescent microparticle immunoassay or syphilis electrochemiluminescence immunoassay) and non‐treponemal (rapid plasma reagin) tests. New syphilis diagnosis was defined as a positive result of both treponemal and non‐treponemal tests without prior treatment or at least a fourfold rise in the non‐treponemal titre for cases with previously treated syphilis. Gonorrhoea and chlamydia testing was done with a nucleic acid amplification test for *Neisseria gonorrhoeae* and *Chlamydia trachomatis* on oral swab, first‐void urine and anal swab samples. All tests were performed using commercially available test kits.

### 2.5 Statistical analysis

Categorical variables are presented as counts and percentages. Continuous variables are presented as medians and interquartile ranges (IQRs). The prevalence of anal HSIL was calculated together with Wilson binomial 95% confidence intervals (CIs). Formal comparisons of continuous and categorical characteristics of participants by the HSIL group were made with a Wilcoxon rank‐sum test and Fisher's exact test, respectively. The incidence of anal HSIL was calculated per person‐time at risk. Person‐time was calculated using the actual participant visit dates. Only the first diagnosed HSIL per participant during the study period was counted as an incident case for calculating HSIL incidence. Histologic anal HSIL was defined as either histologic anal intraepithelial neoplasia (AIN) 2 with positive p16 immunohistochemistry staining or AIN 3. A composite anal HSIL endpoint was defined as a histologic HSIL and/or cytologic HSIL or atypical squamous cells, cannot exclude HSIL. The prevalence and incidence of anal HSIL were calculated. High‐risk anal HPV infection was categorized into three groups: 16, 18/45 and other, based on the categorization limit of the Cepheid Xpert HPV assay (GeneXpert; Cepheid, Sunnyvale, CA) that was used in recent visits by the cohort. The current study does not contain data generated using this assay. The CD4 count was categorized using a cutoff of 350 cells/mm^3^ to represent advanced HIV acquisition [21]. The plasma HIV RNA was categorized based on the median as a cutoff. Factors associated with the prevalence of histologic anal HSIL were assessed using a logistic regression. A threshold of a *p*‐value of <0.2 was used to select variables to adjust for in a multivariable model. Factors associated with the incidence of histologic anal HSIL were assessed using the Kaplan−Meier method and Cox‐proportional hazards regression. A multivariable model was built using variables with *p*<0.2 in the univariable model. Proportional hazards assumption was tested using Schoenfeld residuals. Statistical analysis was conducted with Stata 15 (StataCorp, College Station, TX, USA) and R Statistical Software (v4.1.2).

## 3 RESULTS

### 3.1 Baseline characteristics of participants

A total of 96 cisgender men and TGW who have sex with men with acute HIV acquisition underwent screening from May 2017 to June 2020 (Table 1). One participant was excluded due to valvular heart disease, resulting in the enrolment and follow‐up of 95 participants. Analyses excluded a participant with an abnormal HRA finding at baseline due to the unavailability of a histology sample and a participant whose HIV diagnosis was later determined to be Fiebig stage 6. Consequently, 89 MSM and four TGW were included in the analyses. Of the included participants, 92 were Thai, and one was Filipino. The age at baseline ranged from 18 to 47 years, median 26 (IQR 23–30). The median age of sexual debut was 18 (IQR 16–20). At baseline, more than half of individuals (65.6%) had at least one type of anal high‐risk HPV infection. Among other types, HPV 16, 18 and 45 were present in 15.1%, 7.53% and 6.45% of individuals at baseline, respectively. Only 8/93 (8.6%) of individuals had a baseline CD4 count <200 cells/mm^3^ during acute HIV acquisition. All participants initiated ART, with the median time from acute HIV diagnosis to ART initiation being 3 days (maximum 13). The most common ART regimen was an integrase strand transfer inhibitor‐based regimen (95.7%; Table S1).

**Table 1:** Characteristics of 93 individuals with acute HIV acquisition at study enrolment

		Histologic anal HSIL		
Characteristic	Overall, *N* = 93	No, *N* = 82	Yes, *N* = 11	*p*‐value^a^
Age (years)				0.353
Median (IQR)	26.0 (23.0, 30.0)	27.0 (23.0, 30.0)	24.0 (22.0, 29.0)	
Age group (years)				0.794
<25	34 (36.6%)	28 (34.1%)	6 (54.5%)	
25–29	30 (32.3%)	27 (32.9%)	3 (27.3%)	
30–34	14 (15.1%)	13 (15.9%)	1 (9.1%)	
35–39	8 (8.6%)	7 (8.5%)	1 (9.1%)	
≥40	7 (7.5%)	7 (8.5%)	0 (0%)	
Gender				>0.999
MSM	89 (95.7%)	78 (95.1%)	11 (100.0%)	
TGW	4 (4.3%)	4 (4.9%)	0 (0%)	
Years since first sex				>0.999
0–9	57 (61.3%)	50 (61.0%)	7 (63.6%)	
10–19	26 (28.0%)	23 (28.0%)	3 (27.3%)	
20–29	7 (7.5%)	7 (8.5%)	0 (0%)	
≥30	1 (1.1%)	1 (1.2%)	0 (0%)	
No response	2 (2.2%)	1 (1.2%)	1 (9.1%)	
No. sex partners in lifetime				0.006
1	1 (1.1%)	0 (0%)	1 (9.1%)	
2–5	16 (17.2%)	15 (18.3%)	1 (9.1%)	
6–10	22 (23.7%)	16 (19.5%)	6 (54.5%)	
≥11	54 (58.1%)	51 (62.2%)	3 (27.3%)	
No. receptive anal sex partners in lifetime				0.083
0–1	3 (3.2%)	1 (1.2%)	2 (18.2%)	
2–5	25 (26.9%)	23 (28.0%)	2 (18.2%)	
6–10	18 (19.4%)	15 (18.3%)	3 (27.3%)	
≥11	29 (31.2%)	26 (31.7%)	3 (27.3%)	
No response	18 (19.4%)	17 (20.7%)	1 (9.1%)	
No. receptive anal sex partners in past 6 months				0.261
0–1	18 (19.4%)	15 (18.3%)	3 (27.3%)	
2–5	56 (60.2%)	51 (62.2%)	5 (45.5%)	
6–10	12 (12.9%)	9 (11.0%)	3 (27.3%)	
≥11	7 (7.5%)	7 (8.5%)	0 (0%)	
Sexual position preference				0.888
Insertive only	4 (4.3%)	4 (4.9%)	0 (0%)	
Insertive > receptive	17 (18.3%)	16 (19.5%)	1 (9.1%)	
Insertive = receptive	8 (8.6%)	7 (8.5%)	1 (9.1%)	
Receptive > insertive	30 (32.3%)	25 (30.5%)	5 (45.5%)	
Receptive only	19 (20.4%)	17 (20.7%)	2 (18.2%)	
No response	15 (16.1%)	13 (15.9%)	2 (18.2%)	
Condom use for receptive anal sex in past 6 months				0.341
No receptive in past 6 months	5 (5.4%)	4 (4.9%)	1 (9.1%)	
Never	21 (22.6%)	20 (24.4%)	1 (9.1%)	
Sometimes	62 (66.7%)	54 (65.9%)	8 (72.7%)	
Always	5 (5.4%)	4 (4.9%)	1 (9.1%)	
Syphilis, new diagnosis				>0.999
No	84 (90.3%)	74 (90.2%)	10 (90.9%)	
Yes	9 (9.7%)	8 (9.8%)	1 (9.1%)	
Rectal chlamydia				0.725
No	65 (69.9%)	58 (70.7%)	7 (63.6%)	
Yes	27 (29.0%)	23 (28.0%)	4 (36.4%)	
Not done	1 (1.1%)	1 (1.2%)	0 (0%)	
Rectal gonorrhoea				>0.999
No	74 (79.6%)	65 (79.3%)	9 (81.8%)	
Yes	18 (19.4%)	16 (19.5%)	2 (18.2%)	
Not done	1 (1.1%)	1 (1.2%)	0 (0%)	
Cigarette smoking				0.150
Never	64 (68.8%)	55 (67.1%)	9 (81.8%)	
Former smoker	18 (19.4%)	18 (22.0%)	0 (0%)	
Current smoker	11 (11.8%)	9 (11.0%)	2 (18.2%)	
Anal HPV				>0.999
Negative	20 (21.5%)	18 (22.0%)	2 (18.2%)	
Any low‐risk HPV	10 (10.8%)	9 (11.0%)	1 (9.1%)	
Any high‐risk HPV	61 (65.6%)	54 (65.9%)	7 (63.6%)	
HPV 16^b^ (% of any high‐risk)	14 (15.4%)	11 (13.6%)	3 (30.0%)	0.180
HPV 18 (% of any high‐risk)	7 (7.7%)	7 (8.6%)	0 (0%)	>0.999
HPV 45^b^ (% of any high‐risk)	6 (6.6%)	6 (7.4%)	0 (0%)	>0.999
Invalid	2 (2.2%)	1 (1.2%)	1 (9.1%)	
Fiebig stage				0.064
1	11 (11.8%)	11 (13.4%)	0 (0%)	
2	10 (10.8%)	9 (11.0%)	1 (9.1%)	
3	61 (65.6%)	55 (67.1%)	6 (54.5%)	
4	11 (11.8%)	7 (8.5%)	4 (36.4%)	
5	0 (0%)	0 (0%)	0 (0%)	
Baseline CD4 count (cells/mm^3^)				0.477
<200	8 (8.6%)	8 (9.8%)	0 (0%)	
200–349	39 (41.9%)	32 (39.0%)	7 (63.6%)	
350–499	27 (29.0%)	24 (29.3%)	3 (27.3%)	
≥500	19 (20.4%)	18 (22.0%)	1 (9.1%)	
Plasma HIV RNA (log_10_ copies/ml)				0.146
<4	10 (10.8%)	10 (12.2%)	0 (0%)	
4–4.99	11 (11.8%)	10 (12.2%)	1 (9.1%)	
5–5.99	19 (20.4%)	19 (23.2%)	0 (0%)	
6–6.99	41 (44.1%)	33 (40.2%)	8 (72.7%)	
≥7	12 (12.9%)	10 (12.2%)	2 (18.2%)	

*Note*: Data are presented as *N* (%) or median (IQR).

Abbreviations: HPV, human papillomavirus; HSIL, high‐grade squamous intraepithelial lesion; IQR, interquartile range; MSM, men who have sex with men; TGW, transgender women.

^a^ Not including missing or invalid values in the analysis.

^b^ Two participants positive for both HPV 16 and 45.

### 3.2 Participant retention and follow‐up data

Follow‐up duration from the baseline visit to the last visit ranges from 0 to 46 months (median 33). Cohort retention flowchart is shown in Figure S1. By week 24 of follow‐up, the majority of participants’ CD4 count became ≥350 cells/mm^3^, and all of them were virally suppressed (plasma HIV RNA <1000 copies/ml). Progression of CD4 count and plasma HIV RNA throughout the follow‐up period can be seen in Table S2.

### 3.3 Prevalence and incidence of anal HSIL

Histologic anal HSIL was identified in 11/93 individuals (11.8%) at baseline (Table 2). The prevalence of composite anal HSIL was the same. In the multivariable analysis, only plasma HIV RNA ≥6 log_10_ copies/ml was associated with prevalent anal HSIL adjusted for age (adjusted odds ratio 8.50, 95% CI 1.03–69.93; Table 3).

**Table 2:** Prevalence of anal HSIL and incidence of initial anal HSIL by histologic and composite endpoints

Statistic	Histologic anal HSIL		Composite anal HSIL	
	*n*	% or per 100 PY (95% CI)	*n*	% or per 100 PY (95% CI)
Prevalence (*N* = 93)	11	11.8% (6.1–20.2)	11	11.8% (6.1–20.2)
Incidence (person‐year = 147.0 for histologic and 145.5 for composite)	29	19.7 (13.7–28.4)	30	20.6 (14.4–29.5)

Abbreviations: CI, confidence interval; HSIL, high‐grade squamous intraepithelial lesion; PY, person‐year.

**Table 3:** Uni‐ and multivariable analyses of factors associated with histologic anal HSIL prevalence

Characteristic	Univariable				Multivariable		
	*N*	OR	95% CI	*p*‐value	aOR	95% CI	*p*‐value
Age group (years)	93						
<25		Reference	—			Reference	—
≥25		0.43	0.12–1.54	0.196	0.50	0.13–1.85	0.299
Years since first sex	91						
<10		Reference	—				
≥10		0.69	0.17–2.87	0.611			
No. sex partners in lifetime	93						
1–5		Reference	—				
≥6		1.01	0.20–5.15	0.993			
No. receptive anal sex partners in lifetime	75						
0–5		Reference	—				
≥6		0.88	0.22–3.43	0.852			
No. receptive anal sex partners in past 6 months	93						
0–5		Reference	—				
≥6		1.55	0.37–6.50	0.551			
Condom use for receptive anal sex in past 6 months	93						
No receptive in part 6 months		Reference	—				
Always		0.20	0.01–3.91	0.289			
Never or not always		0.62	0.06–6.20	0.685			
Syphilis, new diagnosis	93						
No		Reference	—				
Yes		0.93	0.10–8.19	0.944			
Rectal chlamydia or gonorrhoea	92						
No		Reference	—				
Yes		0.79	0.21–2.91	0.723			
Cigarette smoking	93						
Never		Reference	—				
Former or current smoker		0.45	0.09–2.24	0.332			
Alcohol drinking	93						
No		Reference	—				
Yes		1.17	0.29–4.78	0.827			
Ever used ATS	93						
No		Reference	—				
Yes		0.54	0.11–2.67	0.448			
Anal HPV	91						
No high‐risk HPV		Reference	—				
High‐risk HPV other than HPV 16		0.84	0.17–4.03	0.825			
HPV 16^a^		2.45	0.43–14.08	0.314			
Fiebig stage	93						
1–3		Reference					
4		6.12	1.43‐26.16	0.014			
CD4 count (cells/mm^3^)	93						
≥350		Reference	—				
<350		1.84	0.50–6.76	0.360			
Plasma HIV RNA (log_10_ copies/ml)	93						
<6		Reference	—			Reference	—
≥6		9.07	1.11–74.13	0.040	8.50	1.03–69.93	0.047

Abbreviations: aOR, adjusted odds ratio; ATS, amphetamine‐type stimulant; CI, confidence interval; HPV, human papillomavirus; OR, odds ratio.

^a^ Two participants positive for both HPV 16 and 45 were grouped in the HPV 16 category.

Of the 82 individuals who did not have anal HSIL at baseline, 80 were followed up during the study period. The time to first incidence of anal HSIL ranged from 5.2 to 44.6 months (median 22.1). The number of person‐years at risk when considering the histologic end point totalled 147.0. During this follow‐up time, 29 out of 80 individuals developed incident histologic anal HSIL. This translates to a 19.7 per 100 person‐years incidence rate of initial histologic anal HSIL (95% CI 13.7–28.4; Table 2).

**Figure 1:**
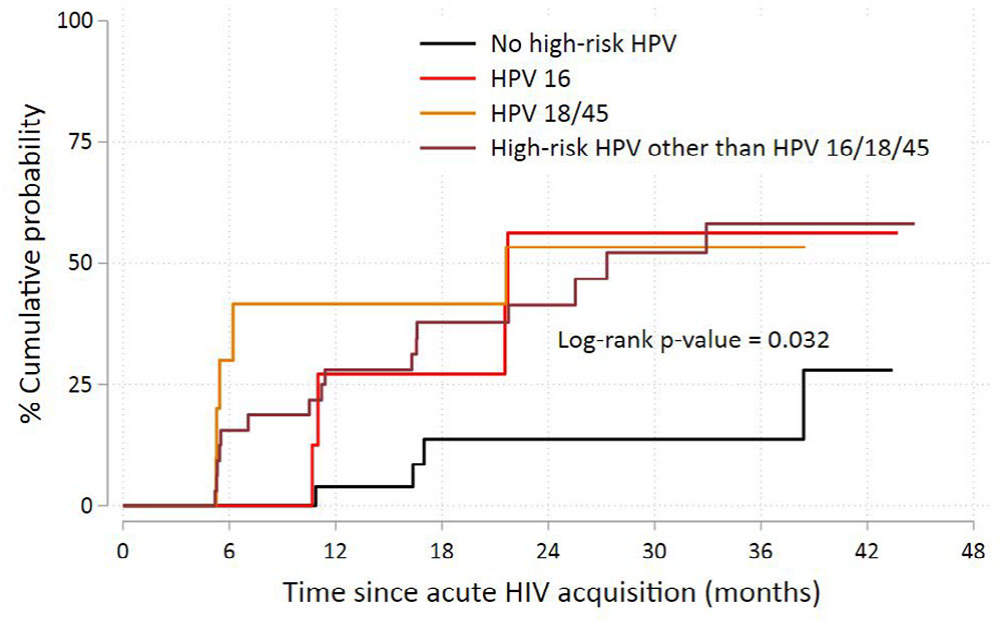
Kaplan−Meier curve of the cumulative probability of initial anal HSIL among participants free of prevalent anal HSIL, stratified by anal high‐risk HPV infection status. *Note*: Median survival of anal HSIL among any baseline anal high‐risk HPV infection was 27.2 months.

Overall, the cumulative probability of incident anal HSIL by month 24 after the diagnosis of acute HIV acquisition was 35.0% (95% CI 25.0%–47.4%), and at month 44 was 48.8% (95% CI 34.0%–66.1%). As shown in Figure 1, there was a significant difference in the probability of initial anal HSIL over time between individuals with and without baseline anal high‐risk HPV infection (*p* = 0.004). Among those with baseline anal high‐risk HPV infection, the median (25th percentile) time from acute HIV acquisition to incident histologic anal HSIL was 27.2 (11.0) months in those with baseline anal high‐risk HPV infection, while among those without baseline high‐risk HPV infection, incident HSIL did not reach 50%, but the 25th percentile was 38.3 months. We found no difference in the probability of anal HSIL over time between individuals infected with anal HPV 16, 18 or 45, and other high‐risk HPV genotypes (log‐rank *p* = 0.833).

In the multivariable analysis, baseline HPV 16 (adjusted hazard ratio [aHR] 4.33, 95% CI 1.03–18.18), HPV 18/45 (aHR 6.82, 95% CI 1.57–29.51), high‐risk HPV other than HPV 16/18/45 (aHR 4.23, 95% CI 1.27–14.14), newly diagnosed syphilis (aHR 4.67, 95% CI 1.10–19.90) and a baseline CD4 count of <350 cells/mm^3^ (aHR 3.09, 95% CI 1.28–7.48) were associated with incident anal HSIL, after adjusting for lifetime number of partners and smoking status (Table 4).

**Table 4:** Uni‐ and multivariable analyses of factors associated with incidence of initial histologic anal HSIL

Characteristic	*N*	Univariable			Multivariable		
		HR	95% CI	*p*‐value	aHR	95% CI	*p*‐value
Age group (years)	80						
<25		Reference	—		Reference	—	
≥25		1.82	0.77–4.26	0.170	1.24	0.48–3.23	0.654
Gender	80						
MSM		Reference	—				
TGW		0.61	0.08–4.50	0.629			
Years since first sex	79						
0–10		Reference	—				
≥10		1.38	0.66–2.87	0.391			
No. sex partners in lifetime	80						
1–5		Reference	—		Reference	—	
≥6		3.22	0.76–13.57	0.112	3.17	0.72–13.89	0.125
No. receptive anal sex partners in lifetime	63						
0–5		Reference	—				
≥6		2.46	0.92–6.57	0.072			
No. receptive anal sex partners in past 6 months	80						
0–5		Reference	—				
≥6		1.44	0.61–3.40	0.399			
Position preference	67						
Insertive		Reference	—				
Receptive or both		1.36	0.18–10.07	0.764			
Syphilis, new diagnosis	80						
No		Reference	—		Reference	—	
Yes		3.36	1.13–10.01	0.030	4.92	1.20–20.23	0.027
Rectal chlamydia or gonorrhoea	79						
No		Reference	—				
Yes		1.29	0.62–2.68	0.488			
Cigarette smoking	80						
Never		Reference	—		Reference	—	
Former smoker		0.66	0.25–1.75	0.408	0.42	0.14–1.24	0.116
Current smoker		0.19	0.03–1.40	0.104	0.22	0.03–1.67	0.143
Alcohol drinking	80						
No		Reference	—				
Yes		1.19	0.52–2.68	0.683			
Ever used ATS	80						
No		Reference	—				
Yes		0.94	0.43–2.07	0.877			
Anal HPV	80						
No high‐risk HPV		Reference	—		Reference	—	
High‐risk HPV other than HPV 16/18/45	79	4.15	1.38–12.52	0.012	4.23	1.27–14.14	0.019
HPV 18/45^a^		5.16	1.38–19.26	0.015	6.82	1.57–29.51	0.010
HPV 16^a^		3.69	0.92–14.81	0.066	4.33	1.03–18.18	0.045
Fiebig stage	80						
1		Reference	—				
2		4.64	1.12–19.13	0.034			
3		1.75	0.50–6.05	0.380			
4		1.85	0.37–9.35	0.456			
CD4 count (cells/mm^3^)	80						
≥350		Reference	—		Reference	—	
<350		2.26	1.05–4.88	0.038	3.09	1.28–7.48	0.012
Plasma HIV RNA (log_10_ copies/ml)	80						
<6		Reference	—				
≥6		1.64	0.77–3.50	0.196			

Abbreviations: aHR, adjusted hazard ratio; ATS, amphetamine‐type stimulant; CI, confidence interval; HPV, human papillomavirus; HR, hazards ratio; MSM, men who have sex with men; TGW, transgender women.

^a^ Two participants positive for both HPV 16 and 45 were grouped in the HPV 16 category.

## 4 DISCUSSION

We demonstrated for the first time the prevalence and incidence of anal HSIL among cisgender men and TGW who have sex with men who were diagnosed and treated during acute HIV acquisition. The anal HSIL prevalence of 11.8% at HIV acquisition and diagnosis was comparable to that of 11.4% in HIV‐negative Thai MSM [10] and a pooled prevalence of 11.3% in a systematic global review of HIV‐negative MSM [12]. However, we observed that the incidence of initial anal HSIL among people who initiated ART during acute HIV acquisition was about midway between those previously reported in HIV‐negative and chronic HIV cohorts. The incidence of initial histologic anal HSIL in the current acute HIV cohort was 19.7 per 100 person‐years, compared with 7.3 and 31.3 per 100 person‐years in Thai MSM without and with HIV, respectively [10]. This finding supports previous evidence that early detection and treatment of HIV in the acute stage of acquisition prevents the immunological dysfunction seen in individuals with chronically acquired HIV [16, 17, 18, 22, 23], potentially protecting against HSIL through the prevention or early resolution of HPV acquisition. However, the incidence of anal HSIL as seen in our study was still higher than that of HIV‐negative individuals.

Our study suggests that using histologic anal HSIL is sufficient as a diagnostic endpoint, as the incremental yield of adding cytologic methods resulted in only one additional diagnosis of HSIL, raising the proportion of the longitudinal cohort diagnosed with HSIL from 29/80 (36.3%) to 30/80 (37.5%). However, this conclusion may not be readily generalizable, as the histologic detection of anal HSIL is very much operator dependent. The ability to detect histologic anal HSIL increases with the experience of the anoscopist [24, 25]. Our anoscopist (NT) had 8 years of experience with HRA at study initiation. In contrast to our findings, another study recommended the use of combined cytologic and histologic anal HSIL endpoints, instead of cytologic or histologic endpoints alone, to capture the majority of anal HSIL [26].

In our cohort, incident histologic anal HSIL was associated with baseline syphilis status, anal high‐risk HPV infection and CD4 count. Infection with anal high‐risk HPV genotypes is indeed the main cause of anal HSIL [4, 5, 8]. Our study demonstrates that anal high‐risk HPV acquisition is associated with a four‐ to seven‐fold increase in the relative incidence of anal HSIL. Thus, HPV vaccines should be given to MSM to prevent anal HPV acquisition [27], preferably before their first sexual encounter. In Thailand, the barrier to receiving HPV vaccines for men is the high price and lack of coverage through the universal health insurance programme. Furthermore, individuals with a lower CD4 count at acute HIV diagnosis were also more likely to develop anal HSIL than those with a higher CD4 count. This indicates that the magnitude of the decline in CD4 count during the acute stage of HIV acquisition, although temporary, has long‐term consequences and is a predictor of future progression to anal HSIL.

The current study has some limitations. Firstly, almost all the study participants were male‐at‐birth Thai people who have had sex with men. Therefore, the findings might not be applicable to other ethnic groups or genders. Secondly, the number of participants was small, since it is uncommon to detect acute HIV acquisition and difficult to recruit people into a study that involves repeated anoscopy and anal biopsy. However, this is the largest cohort to date to study anal HSIL prevalence and incidence among individuals diagnosed and treated during acute HIV acquisition. Thirdly, as discussed earlier, the histologic diagnosis of anal HSIL depends highly on the experience of the operators; hence, the results may vary with other anoscopists. Finally, some variables, such as the number of sexual partners and years since first sex, were based solely on self‐report and were vulnerable to recall bias.

## 5 CONCLUSIONS

With HIV diagnosis and immediate ART initiation during acute HIV acquisition, we found the prevalence of anal HSIL among cisgender men and TGW to be similar to those without HIV. Subsequent anal HSIL incidence, although lower than that of those with chronically acquired HIV, was still higher than that of those without HIV. These findings point to the need to prioritize and integrate anal HSIL screening and management into long‐term care for cisgender MSM living with HIV, regardless of the timing of HIV diagnosis and ART initiation.

## COMPETING INTERESTS

All authors declared no competing interests.

## AUTHORS’ CONTRIBUTIONS

NP, DC and SJK designed the study. NP supervised the study. SN, TC, NC, KP and DC helped with implementation.  NT, PH, AK and JM collected the data. NT performed clinical procedures including anal sample collection, high‐resolution anoscopy and anal biopsy. TP oversaw sample storage and laboratory testing. PM managed study database. SS and ST conducted the primary statistical analysis. ST wrote the manuscript. NP, SS, SN, SJK, RR and CS assisted in the interpretation of the data and provided intellectual input. All authors reviewed and approved the manuscript.

## FUNDING

The project was supported through a grant from amfAR, The Foundation for AIDS Research, with support from the National Institute of Health's National Institute of Allergy and Infectious Diseases, Eunice Kennedy Shriver National Institute of Child Health and Human Development, National Cancer Institute, National Institute of Mental Health, National Institute on Drug Abuse, the National Heart, Lung, and Blood Institute, the National Institute on Alcohol Abuse and Alcoholism, the National Institute of Diabetes and Digestive and Kidney Diseases, and the Fogarty International Center, as part of the International Epidemiology Databases to Evaluate AIDS (IeDEA; U01AI069907).

## DISCLAIMER

The content of this presentation is solely the responsibility of the authors and does not necessarily represent the official views of any of the institutions mentioned above.

## Supporting information

Supporting Information

## Data Availability

The datasets used and/or analysed during the current study are available from the corresponding author on reasonable request.
