# Supplementary material for: Prevalence and incidence of anal high‐grade squamous intraepithelial lesions in a cohort of cisgender men and transgender women who have sex with men diagnosed and treated during acute HIV acquisition in Bangkok, Thailand
Source: J Int AIDS Soc. 2024 May 2;27(5):e26242. doi: 10.1002/jia2.26242 (PMC11064653; doi:10.1002/jia2.26242)

**Supplementary Material**

**Table of Contents**

*Supplementary Tables*

Supplementary Table S1. Additional characteristics of 93 individuals with acute HIV acquisition at study enrollment. Data are presented as N (%) or median (IQR). 1

Supplementary Table S2. Distributions of CD4 count and plasma HIV RNA at baseline and follow-up visits. 2

*Supplementary Figure*

Supplementary Figure S1. Cohort retention flowchart. 3

**Supplementary Table S1. Additional characteristics of 93 individuals with acute HIV acquisition at study enrollment. Data are presented as N (%) or median (IQR).**

| Characteristic | Overall,  N = 93 | Histologic Anal HSIL | | |
| --- | --- | --- | --- | --- |
|  |  | No, N = 82 | Yes, N = 11 | p-value |
| Education |  |  |  | 0.776 |
| Primary School | 2 (2.2%) | 2 (2.4%) | 0 (0%) |  |
| Secondary School | 31 (33.3%) | 26 (31.7%) | 5 (45.5%) |  |
| Technical School or Diploma | 4 (4.3%) | 4 (4.9%) | 0 (0%) |  |
| University or higher | 56 (60.2%) | 50 (61.0%) | 6 (54.5%) |  |
| Occupation |  |  |  | >0.999 |
| Unemployed | 8 (8.6%) | 7 (8.5%) | 1 (9.1%) |  |
| Employed/studying | 85 (91.4%) | 75 (91.5%) | 10 (90.9%) |  |
| Circumcision |  |  |  | 0.350 |
| No | 82 (88.2%) | 71 (86.6%) | 11 (100.0%) |  |
| Yes | 11 (11.8%) | 11 (13.4%) | 0 (0%) |  |
| Alcohol drinking |  |  |  | >0.999 |
| No | 28 (30.1%) | 25 (30.5%) | 3 (27.3%) |  |
| Yes | 65 (69.9%) | 57 (69.5%) | 8 (72.7%) |  |
| ATS use |  |  |  | 0.722 |
| No | 67 (72.0%) | 58 (70.7%) | 9 (81.8%) |  |
| Yes | 26 (28.0%) | 24 (29.3%) | 2 (18.2%) |  |
| ART regimen |  |  |  | >0.999 |
| INSTI-based | 89 (95.7%) | 78 (95.1%) | 11 (100.0%) |  |
| NNRTI-based | 3 (3.2%) | 3 (3.7%) | 0 (0.0%) |  |
| PI-based | 1 (1.1%) | 1 (1.2%) | 0 (0.0%) |  |
| Duration between acute HIV diagnosis and ART initiation |  |  |  | 0.292 |
| Median (IQR) | 3 (2, 3) | 3 (2, 4) | 2 (2, 3) |  |
| Min-Max | 1–13 | 1–13 | 2–5 |  |
| Abbreviations: HSIL, high-grade squamous intraepithelial lesion; ATS, amphetamine-type stimulant; ART, antiretroviral; NSTI, integrase strand transfer inhibitor; NNRTI, nonnucleoside reverse transcriptase inhibitor; PI, protease inhibitor; IQR, interquartile range. | | | | |

**Supplementary Table S2. Distributions of CD4 count and plasma HIV RNA at baseline and follow-up visits.**

| Visit Week | CD4 count (cells/mm^3^) | | Plasma HIV RNA (copies/mL) | |
| --- | --- | --- | --- | --- |
|  | Median (IQR) | Min-Max | Median (IQR) | Min-Max |
| Baseline | 348 (237, 467) | 147–879 | 1.5e6 (2.4e5, 6.9e6) | 71–7.8e7 |
| 24 | 599 (482, 784) | 300–1629 | ≤20 (≤20, ≤20) | 20–128 |
| 48 | 654 (527, 881) | 302–1360 | ≤20 (≤20, ≤20) | 20–795 |
| 72 | 648 (533, 840) | 354–1783 | ≤20 (≤20, ≤20) | 20–50 |
| 96 | 630 (542, 873) | 318–1719 | ≤20 (≤20, ≤20) | 20–398 |
| 120 | 678 (553, 815) | 171–1451 | ≤20 (≤20, ≤20) | 20–58 |
| 144 | 721 (595, 843) | 374–1084 | ≤20 (≤20, ≤20) | 20–83 |
| 168 | 694 (592, 862) | 435–1196 | ≤20 (≤20, ≤20) | 20–62 |
| 192 | 553 (514, 796) | 482–1325 | ≤20 (≤20, ≤20) | 20–24 |
| Abbreviations: IQR, interquartile range. | | | | |

**Supplementary Figure S1. Cohort retention flowchart.**


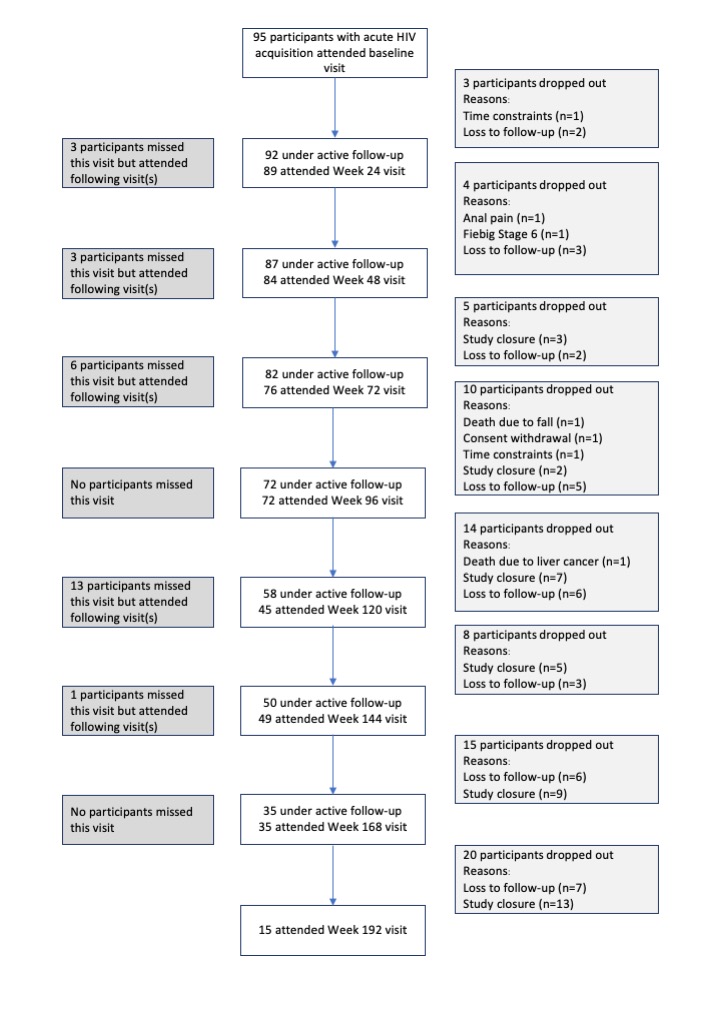

Supplement: Supplementary file 1 — Supporting Information [file JIA2-27-e26242-s001.docx]
